# Giant hepatocellular adenoma as cause of severe abdominal pain: a case report

**DOI:** 10.1186/1752-1947-1-57

**Published:** 2007-07-27

**Authors:** Luigi Sandonato, Calogero Cipolla, Giuseppa Graceffa, Tommaso V Bartolotta, Sergio Li Petri, Oriana Ciacio, Fabio Cannizzaro, Mario A Latteri

**Affiliations:** 1 Department of Oncology, Division of General and Oncological Surgery, University of Palermo, Palermo, Italy; 2 Department of Radiology Interdepartmental Unit for Hepatic Neoplasia Group, University of Palermo, Palermo, Italy

## Abstract

The authors describe the case of a large hepatocellular adenoma diagnosed in a 30-year old woman who came to us complaining of acute pain in the upper abdominal quadrants. The patient had been taking an oral contraceptive pill for the last ten years. We present the clinical features, the diagnostic work-up and the treatment prescribed.

## Background

During the last 30–40 years, there has been an increase in the incidence of hepatocellular adenoma (HCA), a rare primary benign hepatic tumour, in young and middle-aged women, which has been associated with the use of oral contraceptives (OCs) [1].

These tumours may be found accidentally, or they may present with pronounced symptoms, such as acute abdominal pain or haemorrhage due to the rupture of the tumour.

We describe the case of a 30 year-old female patient, who had been taking oral contraceptives for the last ten years and who came to our observation with severe upper abdominal pain as the main symptom of a large HCA.

## Case presentation

A 30-year-old woman was referred to us for the appearance of frequent attacks during the previous week of acute pain in the upper abdominal region; she also complained of nausea and had vomited after eating on several occasions. The patient had no history of abdominal disease and reported that she had been taking a contraceptive pill for the last ten years.

Clinical examination of the abdomen revealed a painful, palpable mass with regular margins of about 6–8 cms. in diameter located in the upper quadrants, between the epigastric region and the left hypochondrium.

Laboratory tests at hospital admittance showed a slight increase in serum transaminase (AST 56 U/1, ALT 73 U/1), whereas haemochrome (RBC 4,70 × 10^6 ^× μl; Hb 12,9 g/dl), γGT, total and fractionated bilirubin, cholinesterase, glycaemia and serum electrolytes were all within normal limits; markers for hepatitis B and C were negative.

A direct X-ray did not show the presence of any abdominal dropsy, while an ultrasound examination (US) revealed a neoformation of about 8 cms in the left hepatic lobe, with clear margins; the mass presented a dyshomogeneous echostructure, with hypoechogenic areas alternating with hyperechogenic zones.

**Figure 1:**
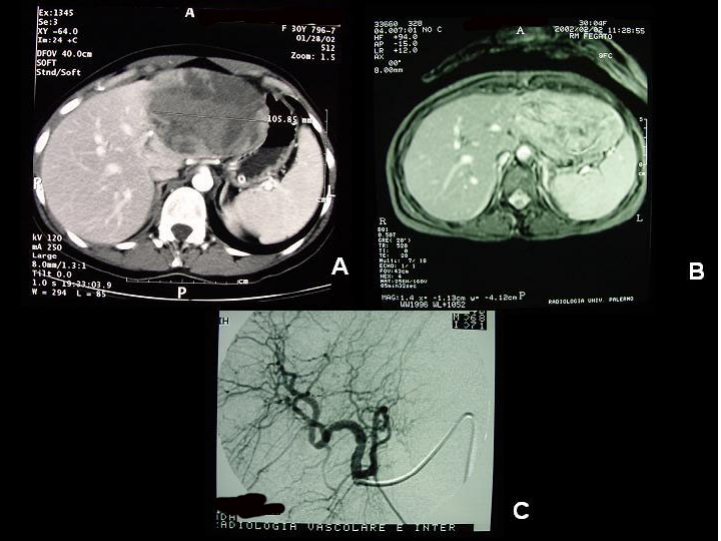
A: Neoformation in the left lobe (CT imaging) B: Neofomation in the left lobe (MR imaging)) C: Angiography: no vision of the left hepatic lobe or neoformation.

An abdominal CT scan using a contrast medium and triphasic techniques, performed 48 hours after admittance, confirmed the presence of a nodular lesion of about 10 cms in diameter in the left lobe of the liver compressing the gastric corpus and fundus but with no signs of infiltration. The lesion showed a dyshomogeneous density with irregular enhancement in the arterial phase and wash-out in the late phase. No infiltrations were observed, either in the parenchyma or within the vascular structure (Fig. 1a).

The diagnostic work-up, together with the medical history of the long-term use of OCs, led us to suspect a large HCA.

We therefore performed a liver biopsy for histopathologic examination which showed a shaft of hepatic tissue containing a few vacuolated hepatocytes within an area of widespread necrosis; no portal or biliary structures were present. Morphological examination of the specimen suggested a diagnosis of hepatic adenoma, although this could not be considered as conclusive.

Four days after admittance, the pain was still present and there was also a reduction of red blood cells and haemoglobin (Rbc 3,69 × 10^6 ^× μl; Hb 9,90 g/dl), together with a further increase in transaminase (AST 156 U/1, ALT 473 U/1) and an increase of LDH (810 IU/l).

Magnetic resonance (MR) was therefore performed with the use of a paramagnetic contrast medium, and this confirmed the presence of a lesion of 12 cms, with clear margins, taking up all the II and III segments of the liver (Fig. 1b) and also another nodule of 1 cm with the same features in the VI segment. Several interlesional areas showing up as spontaneously hyperintense in the T1-w sequences indicated probable recent bleeding.

Not only was the tumour extremely large and causing considerable pain, most probably due to the distension of Glisson's capsule, but laboratory tests and imaging also revealed endotumoral bleeding, indicating a probable rupture of the neoplasia, and we thus decided that surgery was indicated.

At this point it was thus decided to perform an angiographic study in order to check hepatic vascularisation. Catheterisation of the coeliac tripod, performed during preoperative angiographic examination of the hepatic vascularisation, showed the presence of a large dyshomogeneously hypervascular neoformation within the left hepatic lobe associated with the presence of an anatomic variant in which the left hepatic artery originated from the right gastric artery. Selective catheterisation of the common hepatic artery did not reveal any opacity within the neoformation (Fig. 1c).

**Figure 2:**
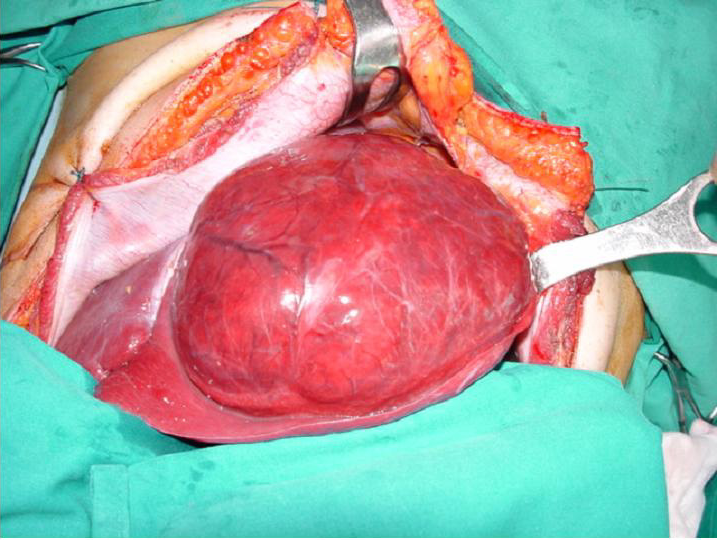
Intraoperative view of the tumour.

**Figure 3:**
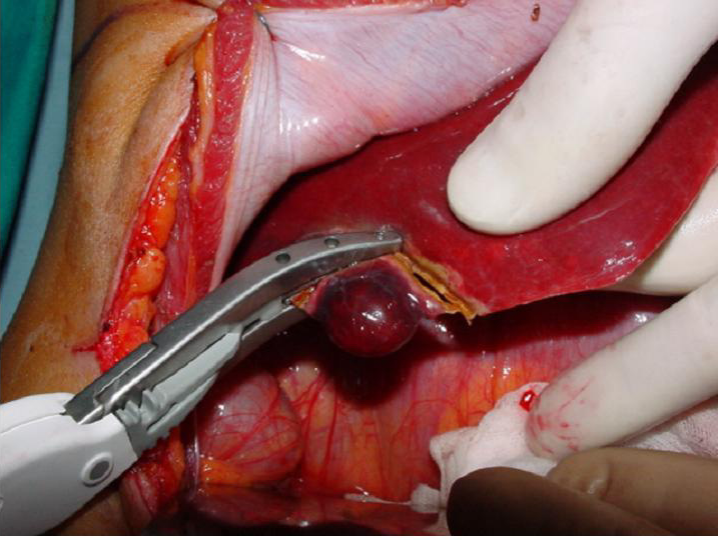
Removal of the nodule from the VI segment.

The patient therefore underwent left hepatic lobectomy (Fig. 2) and focal resection of about 1.5 cms of the VI segment (Fig. 3). Intraoperative ultrasound did not reveal any further lesions. There were no post-operative complications and the patient was sent home on Day VII.

The anatomopathological examination showed a mass of 11.5 cms with a smooth, regular external surface and well-defined margins; the walls were of a yellowish colour and in the centre there was a wide area of necrotic, haemorrhagic tissue extending almost as far as Glisson's capsule. Microscopic examination showed the presence of mature, vacuolated hepatocytes; no portal or biliary structures were present, which confirmed the diagnosis of hepatic adenoma. The smaller nodule in the VI segment presented exactly the same histopathological features.

Following surgery, the patient stopped taking OCs and ultrasound follow-up examination at six months did not reveal the presence of any further focal lesions.

## Conclusion

HCA is a primary benign tumour of hepatocellular origin rarely seen before the introduction of OCs in the 1960s [1]. In 1973, Baum et al were the first to suspect a link between HCA and use of OCs [2]. More often than not, patients with HCA have no symptoms and present a normal liver function with no rise in alpha-foetoprotein serum level. Large adenomas, however, may cause anaemia because of tumoral bleeding, or pain in the upper abdominal quadrants with abdominal distress, and may lead to spontaneous rupture or haemorrhage and, in certain rare cases, even death.

Several diagnostic procedures, such as US, CT and MR, can indicate the presence of an HCA, but this diagnosis must be confirmed by the histopathological examination.

Most HCAs are usually first detected at US. The US HCA may appear as a hyperechoic lesion or else as a hypo- or anechoic solid mass, well-circumscribed and rarely capsulated. A mixed appearance is typical of voluminous and dyshomogeneous masses presenting haemorrhage or necrosis; calcifications are rarely present.

Colour Doppler US may provide some clues for distinguishing HCA from FNH, since the former shows a continuous venous flow in the central portion and either a pulsatile or continuous peripheral flow ('basket pattern'). These findings are absent in FNH, in which colour Doppler US may show a typical spoke-wheel arterial pattern of vessels [3].

On pre-contrast CT scans, HCA has a varied and non-specific appearance, possibly with hypoattenuating areas due to the presence of fat, previous haemorrhage or necrosis, whereas recent haemorrhage or large amounts of glycogen are observed as hyperdense areas. On contrast CT, HCA often shows substantial enhancement during the arterial phase, decreasing during the portal phase and gradually becoming iso- or hypodense in the liver on delayed scans. In some cases, there may be a thin, continuous hyperdense rim due to the presence of a peripheral capsule [4].

HCAs frequently show heterogeneous hypointensity both on un-enhanced T1-weighted and T2-weighted images due to the presence of fat, haemorrhage, or necrosis [5]. Sometimes, a peripheral rim, corresponding histologically to a pseudocapsule, is seen as a low signal-intensity rim on both T1-weighted and T2-weighted images [6,7]. After the administration of gadolinium-chelates, most HCAs show intense enhancement in the arterial phase and are isointense in liver tissue on portal-venous and equilibrium images [8]. Hepatocellular-specific contrast agents may provide useful clues for distinguishing HCA from FNH. After the injection of such an agent, for example gadolinium benzyloxypropionictetraacetate (Gd- BOPTA), HCA typically appears hypointense on delayed phase imaging, due to the lack of biliary ducts, whereas FNH generally appears isointense or slightly hyperintense. When reticuloendothelial cell-targeted contrast agents are used, such as ferumoxides (superparamagnetic iron oxides), some HCAs may show some signal intensity loss, which might be explained by the presence of Kupffer cells in the lesions.

The various imaging techniques, which are extremely important for the diagnosis of HCA, are particularly useful in all those cases where pain is among the symptoms. The lesion should be kept under strict observation and any rapid increase in volume with endotumoral bleeding linked to a reduction of haemochrome parameters should suggest an immediate surgical approach in order to avoid complications which might be brought about by possible rupture of the tumour.

In the case observed by us, the US and the CT scan using a contrast medium and triphasic techniques were essential for reaching a diagnosis. The ultrasound and radiographic features of the lesion, correlated to a medical history of the long-term use of Ocs, made it possible right from the beginning of the diagnostic work-up to suspect the presence of an HCA. Nevertheless, we considered that this diagnostic hypothesis should be confirmed by means of a biopsy, since, although the risk of malignant transformation of an HCA is fairly low, it may occur, and would be an important indication for a surgical approach. In any case, this treatment is not particularly invasive, is well-tolerated and does not generally involve a high rate of complications.

During hospitalisation, an MR examination performed because the patient was becoming progressively more and more anaemic showed a further increase in the size of the lesion and probable endotumoral bleeding. Since this fact indicated a surgical approach due to the high risk of endoperitoneal rupture of the lesion, we immediately performed selective angiography of the liver in order to evaluate vascularisation of the organ and of the tumour. This examination revealed the presence of an anatomic variant in which the left hepatic artery originated from the right gastric artery, and showed that the neoplasia was vascularised by the left hepatic artery.

The therapeutic approach to HCA is still not clear. For asymptomatic patients, conservative treatment requires stringent follow-up with ultrasonography of the liver and only in the case of further growth is surgical treatment indicated. In a recent review regarding the indications for a surgical approach towards benign hepatic neoplasias[9], it has been pointed out that no randomised clinical trials have ever been conducted and that most published reports involve a small number of cases of various types of tumours. Vast, long-term randomised clinical trials with adequate methodology are need for a valid assessment of the advantages and disadvantages of elective surgery for benign liver tumours.

The role of elective surgical resection for HCA is still controversial and mainly depends upon the risk of complications, the uncertain diagnosis and the presence of symptoms related to tumour size and site, particularly with regard to the risk of rupture and resulting haemorrhage [1,9]. Elective resection of HCA has a mortality rate of less than 1%, whereas the mortality rate with free rupture is 5 to 10%.

In the case observed by us, the persistent pain caused by the tumoral growth and the resulting distension of Glisson's capsule, together with progressive anaemia brought about by endotumoral bleeding, led us to suspect the possible rupture of the tumour with consequent haemoperitoneum; immediate surgery was thus considered the treatment of choice.

In conclusion, HCA is a rare benign tumour of the liver, generally involving a history of a prolonged use of OCs. Accurate diagnostic imaging almost always provides a correct differential diagnosis from other benign tumours of the liver. Although there is still some doubt regarding the therapeutic approach to asymptomatic patients, surgery is probably indicated in large-size HCAs with or without abdominal symptoms in order to avoid certain complications such as haemorrhage or rupture of the tumour.

## Competing interests

The author(s) declare that they have no competing interests.

## Authors' contributions

LS, CC, and GG performed the operation, assessed the didactic importance of the clinical case and therefore acquired the data; TVB and FC were responsible for the imaging; SLP and OC. TVB and FC were involved in drafting the manuscript and its revision; MAL gave the final approval of the version to be published.

All authors read and approved the final manuscript.
